# Biological aspects of the lingual papillae of the Arab Zebu cattle: a new perspicuity of its chad ecological adaptations

**DOI:** 10.1186/s40850-024-00208-w

**Published:** 2024-08-12

**Authors:** Mohamed Abumandour, Seham Haddad, Foad Farrag, Ramadan Kandyel, Karam Roshdy, Diaa Massoud, Eman Kamal Khalil

**Affiliations:** 1 Department of Anatomy and Embryology, Faculty of Veterinary Medicine, Alexandria University, Abees 10th, Alexandria, 21944 Egypt; 2 Department of Anatomy and Embryology, Faculty of Veterinary Medicine, University of Sadat City, Sadat City, 32897 Egypt; 3 Department of Anatomy and Embryology, Faculty of Veterinary Medicine, Kafrelsheikh University, Kafrelsheikh, 33511 Egypt; 4 Department of Zoology, Faculty of Science, Tanta University, Tanta, Egypt; 5 Department of Biology, Faculty of Arts and Sciences, Najran University, Najran, Saudi Arabia; 6 Department of Histology and Cytology, Faculty of Veterinary Medicine, Alexandria University, Alexandria, Egypt; 7 Department of Zoology, Faculty of Science, Fayoum University, Fayoum, Egypt; 8 Department of Biology, College of Science, Jouf University, P.O. Box 2014, Sakaka, Al-Jouf, Saudi Arabia; 9 Department of Anatomy and Embryology, Faculty of Veterinary Medicine, Benha University, Benha, Egypt

**Keywords:** Arab zebu cattle, Papillary system, Lingual scales, Filiform papillae, Scanning electron microscope (SEM)

## Abstract

**Background:**

Our research is the first to explore the ultrastructural features of the lingual papillary system of Arab Zebu cattle, highlighting their Chadian environmental adaptations.

**Results:**

There were two types of papillary systems: gustatory (fungiform and circumvallate) and mechanical (filiform, conical, and lentiform). The dorsal surface of the apex and rostral parts of the body had well-developed filiform papillae, whereas the tip’s surface had mucosal folds, tubercles, and few filiform papillae. The torus lingua’s dorsal surface displayed few lentiform papillae, while two conical papillae subtypes and numerous circumvallate papillae were present on its lateral surfaces. A slight median ridge on the dorsal surface of the body had not been described previously. Six filiform papillae subtypes were identified: long and rod-like on the tip; tongue-like and elongated on the lateral area of the apex and body; transient conical and leaf-like on the median line. The accessory processes were: one pair (on long, tongue-like, and transient conical), two pairs (on leaf-like and elongated), and four pairs on the large conical papillae. The two fungiform papillae subtypes were surrounded by a groove and had taste pores (3–5 on the oval and 5–9 on the round papillae). The U-shaped annular bad were observed around the ovoid circumvallate papillae, and the circular bad were observed around the round ones. The circumvallate had taste pores (8–14 on the round’s dorsal and lateral surfaces and 6–10 on the ovoid’s lateral surface).

**Conclusion:**

The papillary system’s regional divergence was specialized for its harsh and semi-harsh diet.

## Background

Arab Zebu cattle, also known as Arab Shuwa, Arab Choa, or Wadera cattle, are a type of cattle originating from traders in Chad impacted by the North Sudan Zebu and Fulani in Cameroon, affecting their widespread distribution. Their color is often chestnut or dark red-and-black, with or without little white patches on the underside, and is less frequently reddish-brown or black [1]. The Arab Zebu cattle of *Bos indicus* have belonged to the *Bos* Genus, *Bovini* Tribe, *Bovinae* subfamily, *Bovidae* family, and *Artiodactyla* order. They can tolerate extreme heat, ticks, insect bites, and a lack of water and food. This breed was utilized by nomadic herders in the arid Sahel region for milk and meat production [2, 3]. Chad’s main income source is cattle ranching, especially in the Savanna Forest, where cattle have adapted to harsh conditions. These cattle can chase pastures for long distances and withstand the hot, tropical climate. The North’s driest region receives little rain, unlike the southern and central regions, which have rainy seasons [4]. Chad has a dry season for the most part and is frequently windy.

Nutritional and dietary systems are crucial for animals’ adaptation to changing environments [5]. The tongue plays a significant role in feeding mechanisms, from particle selection to soft bolus transformation and passage to the esophagus. Previous research had primarily focused on describing the gross, histological, and SEM aspects of the animal tongue to evaluate its adaptations to different environmental conditions [6, 7]. The dorsal lingual mucosal surface contains various papillae that support the gustatory or mechanical role in the specific feeding strategies of mammals, with the tongue acting as a reflector for lingual structural indications of changes in lifestyles [8, 9].

The present work is the first to describe the ultrastructural features of the lingual papillary system of Arab Zebu cattle in Chad, illustrating their adaptations to the harsh desert environmental conditions. The research aimed to analyze the scanning electron microscopic characterizations of the lingual papillary system of Arab Zebu cattle (Bos indicus) and their adaptation to Chad’s Savanna Forest’s harsh desert environment, and compare their findings to those of other ruminant species in similar or different habitats. These lingual adaptations may offer insights into the evolutionary mechanisms that have enabled it to thrive in challenging environments.

## Methods

### Sample’s collection

Eight tongues of both sexes of mature Arab Zebu cattle (4 to 5 years old) with no history of tongue injuries or abnormalities were collected from the N’Djamena slaughterhouse in Chad by the veterinarian in a local slaughterhouse. The age of the examined Arab Zebu cattle was determined according to Best [10]. The animals were slaughtered for meat consumption, not for experimental research purposes. The tongues were collected at the slaughterhouse, placed on ice, and immediately transferred to the laboratory.

### Gross morphology observations

The collected tongues were examined to show the tongue’s general anatomical features, including its papillary system. Following that, the samples were preserved in a 10% formalin solution (Al Mottahedoon Pharma©) for 24–48 h, then transferred four tongues to the fixative solution for scanning electron microscopy, according to Gewily, et al. [11], Kandyel, et al. [12], while the other four were used for the gross anatomical examinations and photographed using a digital camera (*Canon IXY 325*,* Japan*). The anatomical nomenclature was applied according to *Nomina Anatomica Veterinaria* [13].

### For morphometric analysis

The electronic ruler, which has 0.1 mm accuracy, and a camera (*IXY 325*,* Canon*,* Japan*) were used to measure the proportions of the mature Arab Zebu cattle tongue. These measurements were taken to compare the tongue’s relative length and width of the different lingual parts (apex, body, torus linguae, and root). We used the Image J program of the SEM images to calculate the average dimensions of the different lingual papillae on the dorsal surface of the lingual body and the paralingual conical papillae (on the floor of the oral cavity) by (um).

### For scanning electron microscopy (SEM)

Four tongues were prepared for the SEM application according to Farrag, et al. [14]. The samples were fixed at 4 °C in a fixation solution containing 2% formaldehyde and 1.25% glutaraldehyde in a pH 7.2 0.1 M sodium cacodylate buffer. After fixation, the samples were rinsed in 0.1 M sodium cacodylate containing 5% sucrose and then treated with tannic acid. Finally, the lingual samples were dehydrated in increasing ethanol concentrations for 15 min each (in 50, 70, 80, 90, 95, and 100% ethanol). The samples were then dried in carbon dioxide and bonded to stubs using colloidal carbon before being sputter-coated with gold-palladium. Finally, a JEOL scanning electron microscope was used to inspect and photograph the obtained samples.

### Digital coloring of scanning electron microscopic images

We digitally colored the SEM images using the Photo Filter 6.3.2 program to identify the various structures. This technique was previously described by Roshdy, et al. [15].

## Results

### I- gross analysis

**Fig. 1:**
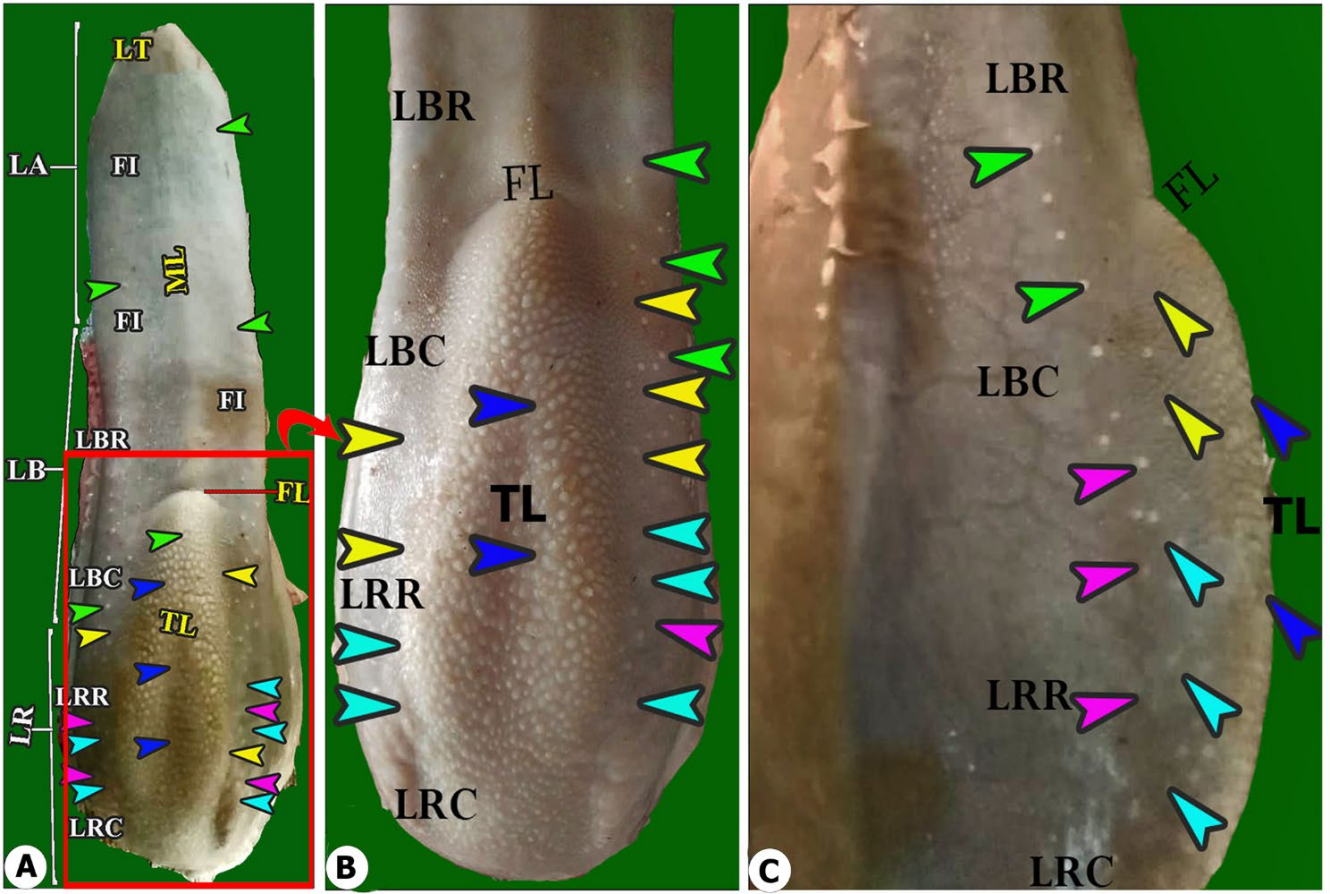
Gross anatomical image of the tongue of the Arab Zebu cattle to show; the apex (LA) with its tip (LT); body (LB) with its rostral (LBR) and caudal (LBC) part; root (LR) with its rostral (LRR) and caudal (LRC) parts; median ridge (ML); fossa lingua (FL); torus lingua (TL) with their small (turquoise arrowheads) and large (yellow arrowheads) conical; circumvallate (purple arrowheads) papilae on its lateral surfcae and lentifrom (blue arrowheads); fungifrom (green arrowheads) on its dorsal surface; the filiform papillae (FI);

**Fig. 2:**
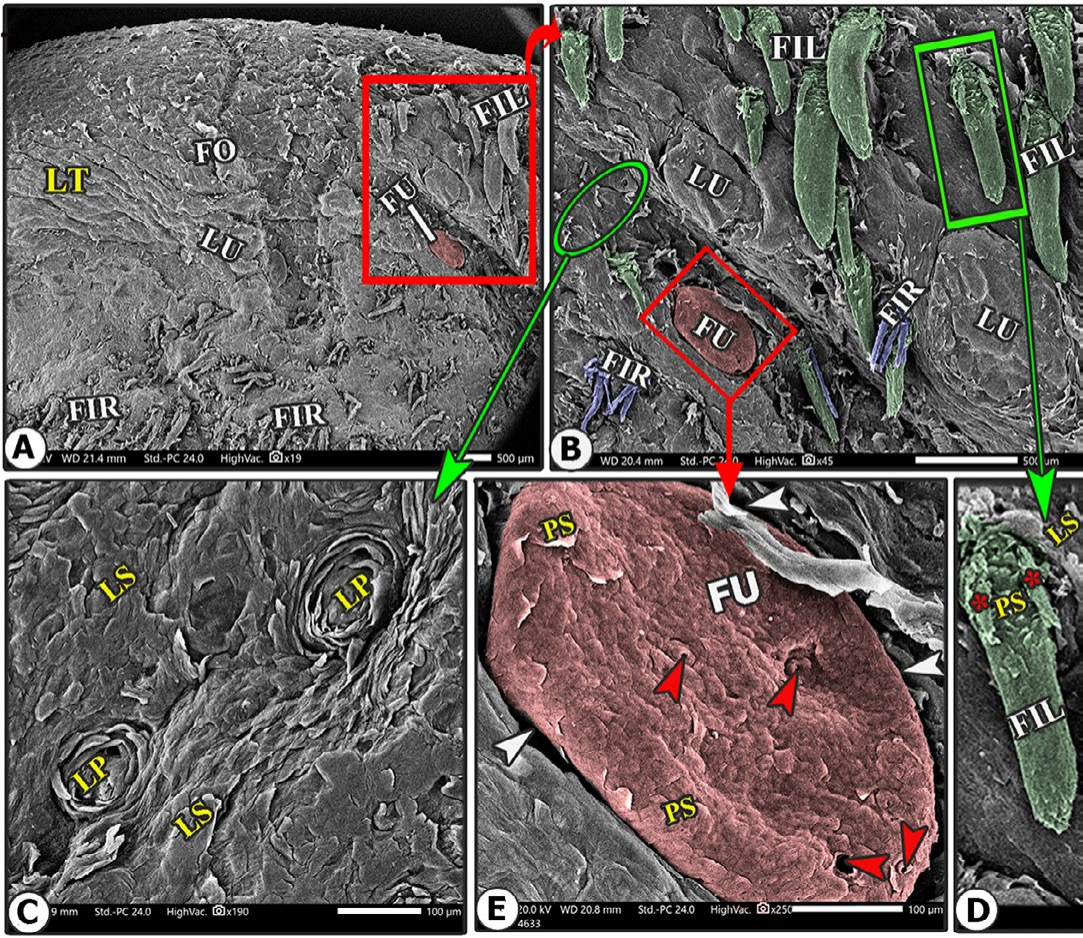
Scanning electron microscopic image of the lingual tip (LT) of the Arab Zebu cattle to show mucosal folds (FO), tubercles (LU), scales (LS), and scale-projections (LP); the filiform papillae (FI); long filiform papillae (FIL) with their accessory processes (red*) and papillary scales (PS); rod-like filiform papillae (FIR); fungiform papillae (FU) with its papillary scales (PS); annular groove (white arrowheads); and taste pores (red arrowheads). Scale bars: A and B = 500 μm, C, D, and E = 100 μm

**Fig. 3:**
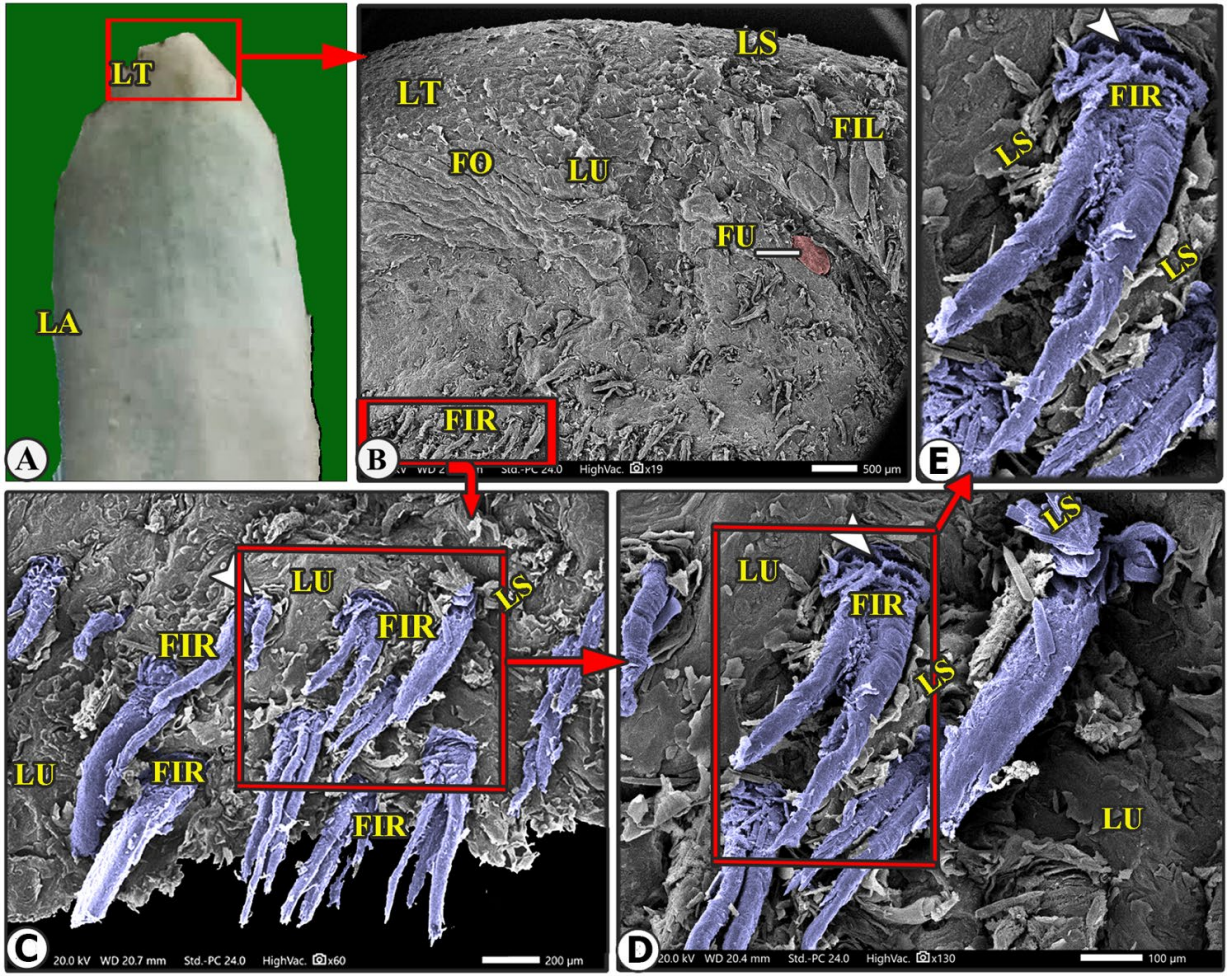
Gross (View A) and scanning electron microscopic (Views B-F) image of the lingual apex (LA) of the Arab Zebu cattle to show the lingual tip (LT) with its mucosal folds (FO); tubercles (LU); scales (LS); a few long filiform papillae (FIL); rod-like filiform papillae (FIR) that are surrounded by a circular groove (white arrowheads); and fungiform papillae (FU). Scale bars: B = 500 μm, C and E = 200 μm, D = 100 μm

**Fig. 4:**
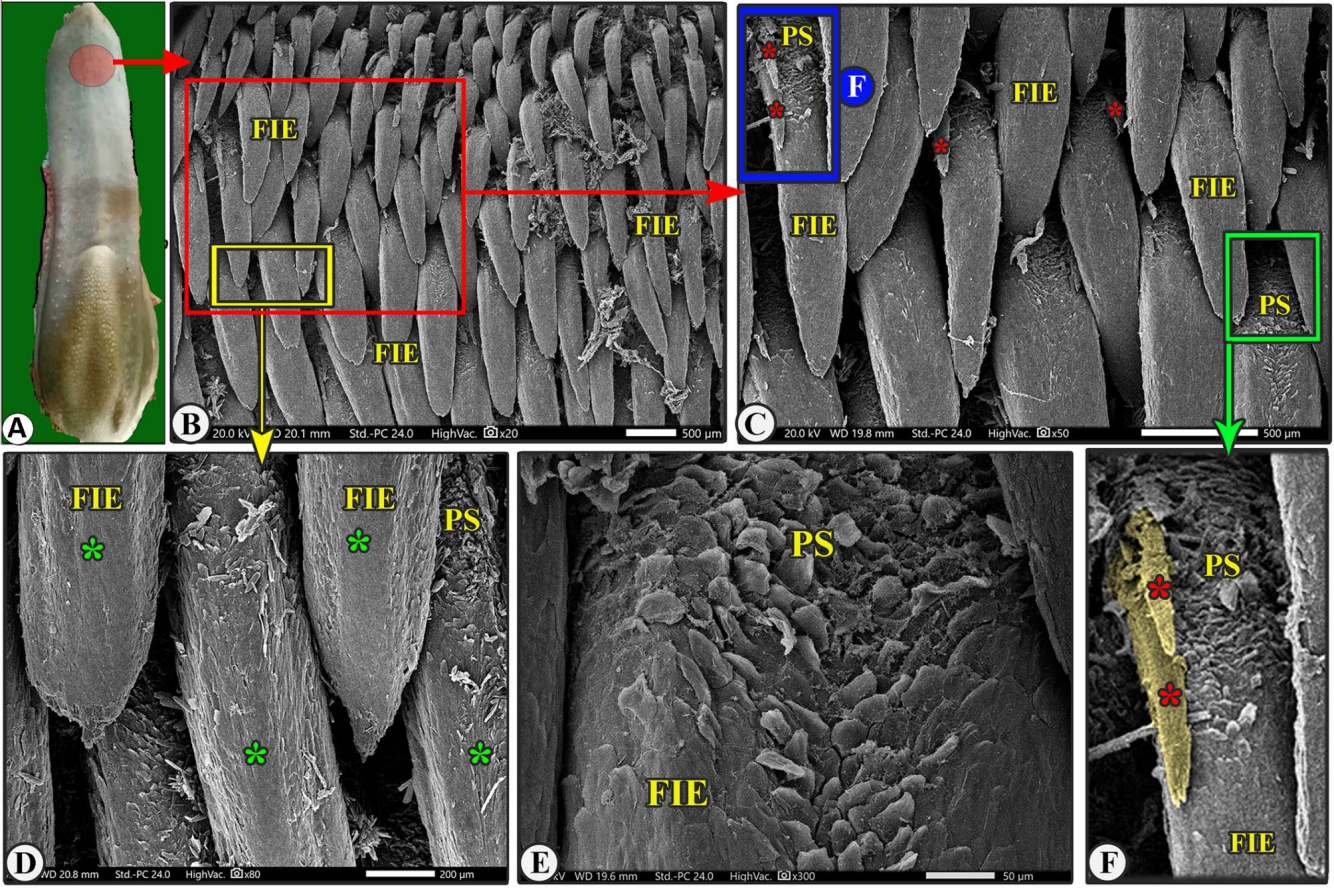
Gross (View A) and scanning electron microscopic (Views B-F) image of the median area of the lingual apex of the Arab Zebu cattle to show the elongated filiform papillae (FIE) with their accessory processes (red*), papillary scales (PS), and median papillary ridge (green*). Scale bars: B and C = 500 μm, D and F = 200 μm, E = 50 μm

The floor of the oral cavity was occupied by an elongated tongue that was divided into three main parts: the apex, the body, and the root. The tongue was classified according to its motility into the anterior motile part (including the apex and the rostral part of the lingual body) and the caudal fixed part (including the caudal part of the body and the lingual root), as described in (Figs. 1, 2A and 3A). The apex had a round tip, two lateral areas, and the median area (Fig. 1), whereas the body was differentiated into the rostral and caudal parts by the fossa linguae (Fig. 1), and the root was subdivided into rostral papillary and caudal non-papillary areas. The torus linguae formed from the caudal part of the body and the rostral part of the root; additionally, it was subdivided into two parts: the rostral triangular part (caudal half of the body) and the caudal wide quadrilateral part (rostral part of the root), as seen in (Figs. 1, 2A, 3A and 4A). The caudal part of the root was devoid of any lingual papillae. The lingual frenulum connected the body’s ventral surface to the floor of the oral cavity.

The gross morphometric analysis in (Table 1) revealed that the tongue length was about 25.65 ± 1.03 cm, and the lingual apex represented 33.2% of total lingual length that measured about 8.52 ± 0.12 cm, while the lingual body represented 47.6% of total lingual length that measured about 12.23 ± 0.37 cm, and the lingual root represented 16% of total lingual length that measured about 4.9 ± 0.24 cm. The torus linguae represented 20.3% of the total lingual length, which measured about 4.12 ± 0.08 cm. Moreover, Table 1 revealed the high number of circumvallate papillae that reached about 25–27 pairs, with about 13–14 pairs forming the dorsal papillary row and 12–13 pairs forming the ventral papillary row. The tongue was wider at its apex, which measured about 6.45 ± 0.54 cm, but less wider at its root, which reached 3.78 ± 0.17 cm.

**Table 1:** Shows the average dimensions of the various lingual parts (apex, body, torus linguae, and root) of the Arab Zebu cattle tongue

Dimensions of tongue				Means ± SD (cm)		
**Tongue**		**Length**		25.65 ± 1.03		
**Lingual apex**		**Length**		8.52 ± 0.12		
		**Width (at its middle part)**		**6.45 ± 0.54**		
		**Thickness (at its middle part)**		**5.23 ± 0.87**		
**Lingual body**		**Length**		**12.23 ± 0.37**		
		**Width (at the level of 2nd premolar teeth)**		**6.21 ± 0.82**		
		**Width (at the level of fossa linguae)**		**6.27 ± 0.56**		
		**Width (at the level of the glossopalatine arch)**		**6.31 ± 0.23**		
**Lingual root**		**Length**		**4.9 ± 0.24**		
		**Width**		**3.78 ± 0.17**		
**Torus linguae**		**Length**		**4.12 ± 0.08**		
		**Width (at middle part)**		**5.21 ± 0.34**		
**Circumvallate papillae**		**Total Number (pairs)**		**25–27**		
		**Number of papillae on dorsal row**		**13–14**		
		**Number of papillae on ventral row**		**12–13**		

### II- scanning electron microscopic (SEM) observations

**Fig. 5:**
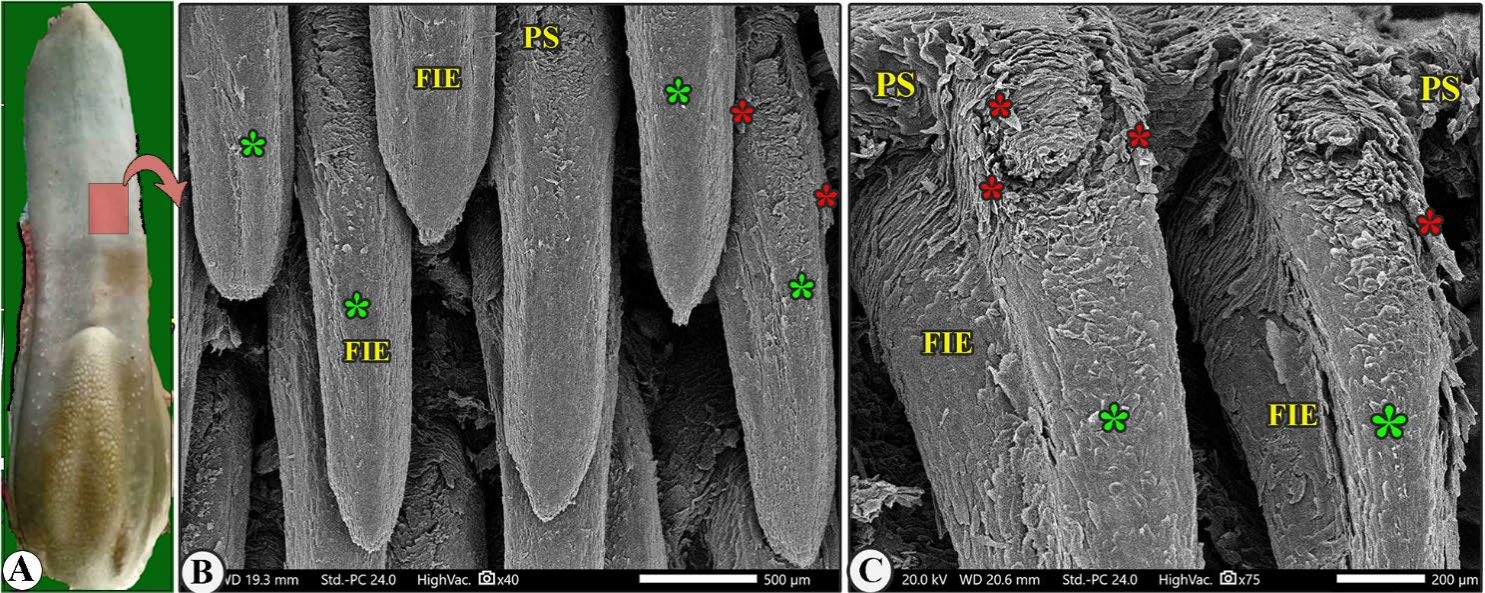
Scanning electron microscopic image of the median area of the lingual apex (LA) of the Arab Zebu cattle to show the elongated filiform papillae (FIE) with their accessory processes (red*), papillary scales (PS), and median papillary ridge (green*). Scale bars: B = 500 μm, C = 200 μm

**Fig. 6:**
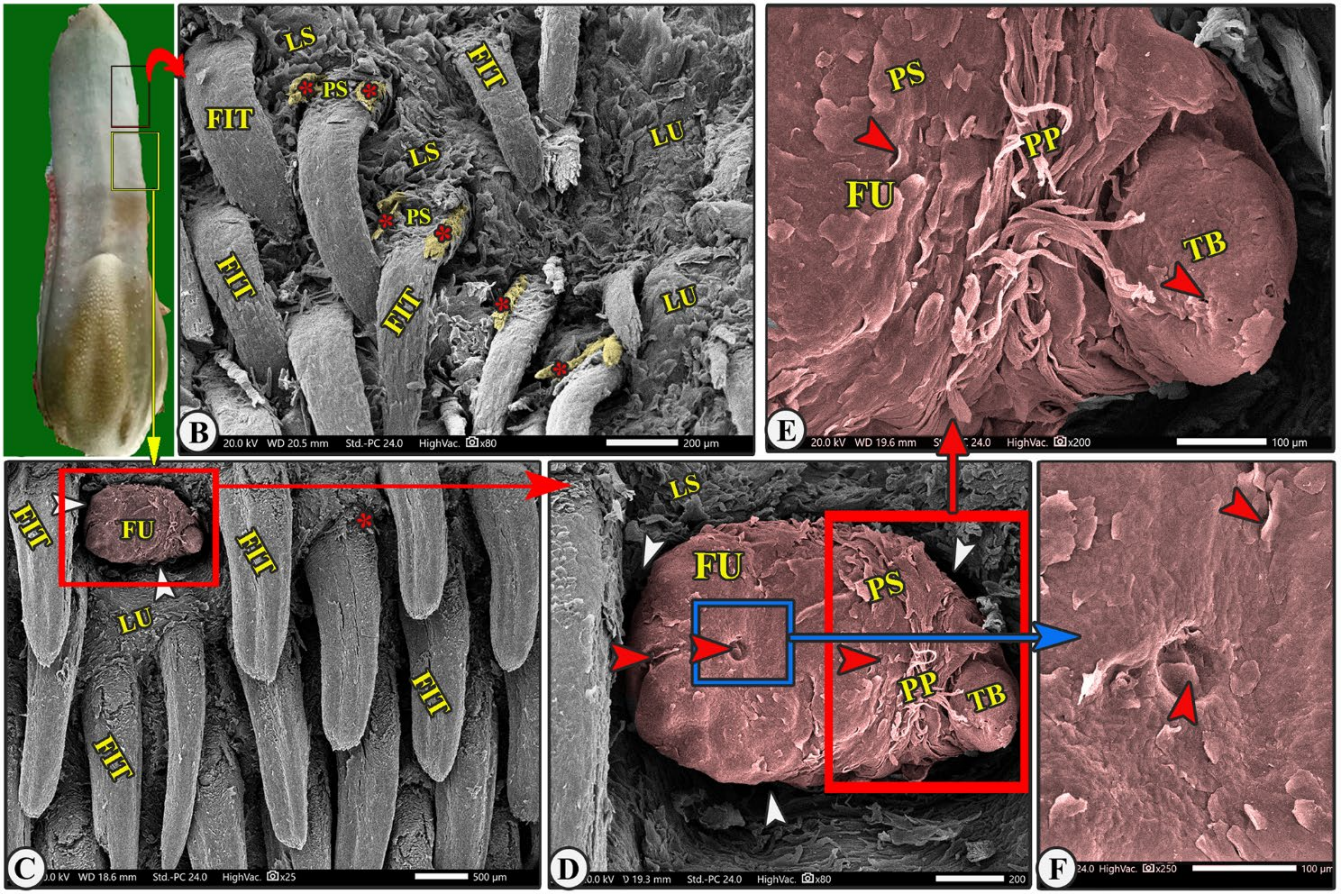
Gross (View A) and scanning electron microscopic (Views B-F) image of the lateral area of the lingual apex (LA) of the Arab Zebu cattle to show the tongue-like filiform papillae (FIT) with their accessory processes (red*), papillary scales (PS), lingual scales (LS), tubercles (LU), fungiform papillae (FU) with their surrounded groove (white arrowheads), taste pores (red arrowheads), papillary scales (PS), taste bud (TB), and papillary processes (PP). Scale bars: B and D = 200 μm, C = 500 μm, E and F = 100 μm

**Fig. 7:**
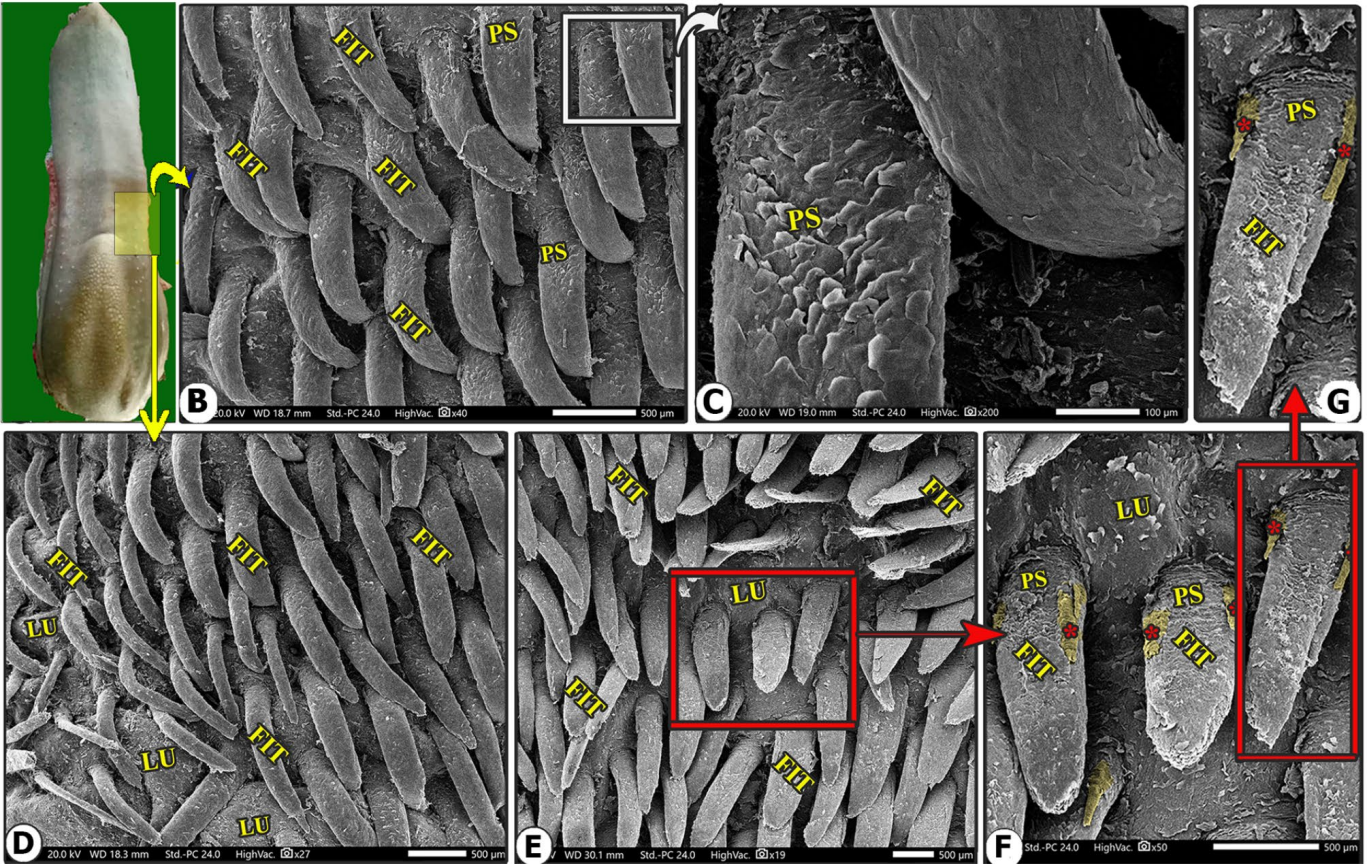
Gross (View A) and scanning electron microscopic (Views B-F) image of the lateral area of the lingual body (LB) of the Arab Zebu cattle to show the tongue-like filiform papillae (FIT) with their accessory processes (red*), papillary scales (PS), and lingul tubercle (LU). Scale bars: B, D, E, and F = 500 μm, C = 100 μm, G = 50 μm

**Fig. 8:**
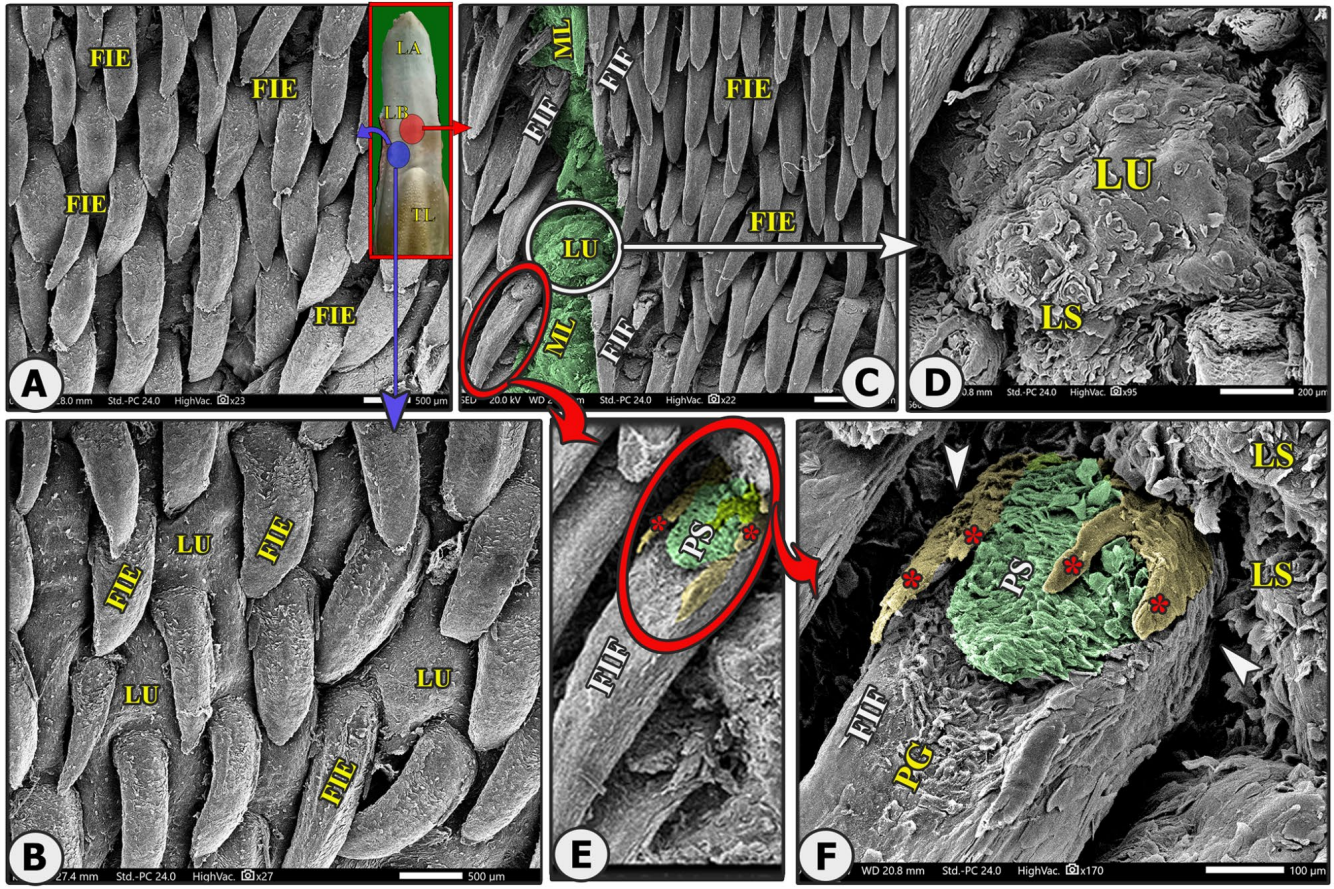
Gross (View A) and scanning electron microscopic (Views B-F) image of the median area of the lingual body (LB) of the Arab Zebu cattle to show; the apex (LA), torus lingua (TL), the elongated filiform papillae (FIE), leaf-like filiform papillae (FIE) with their accessory processes (red*), papillary scales (PS), median papillary groove (PG), the median ridge (ML), lingual tubercle (LU), and scales (LS). Scale bars: A, B, and C = 500 μm, D = 200 μm, E and F = 100 μm

**Fig. 9:**
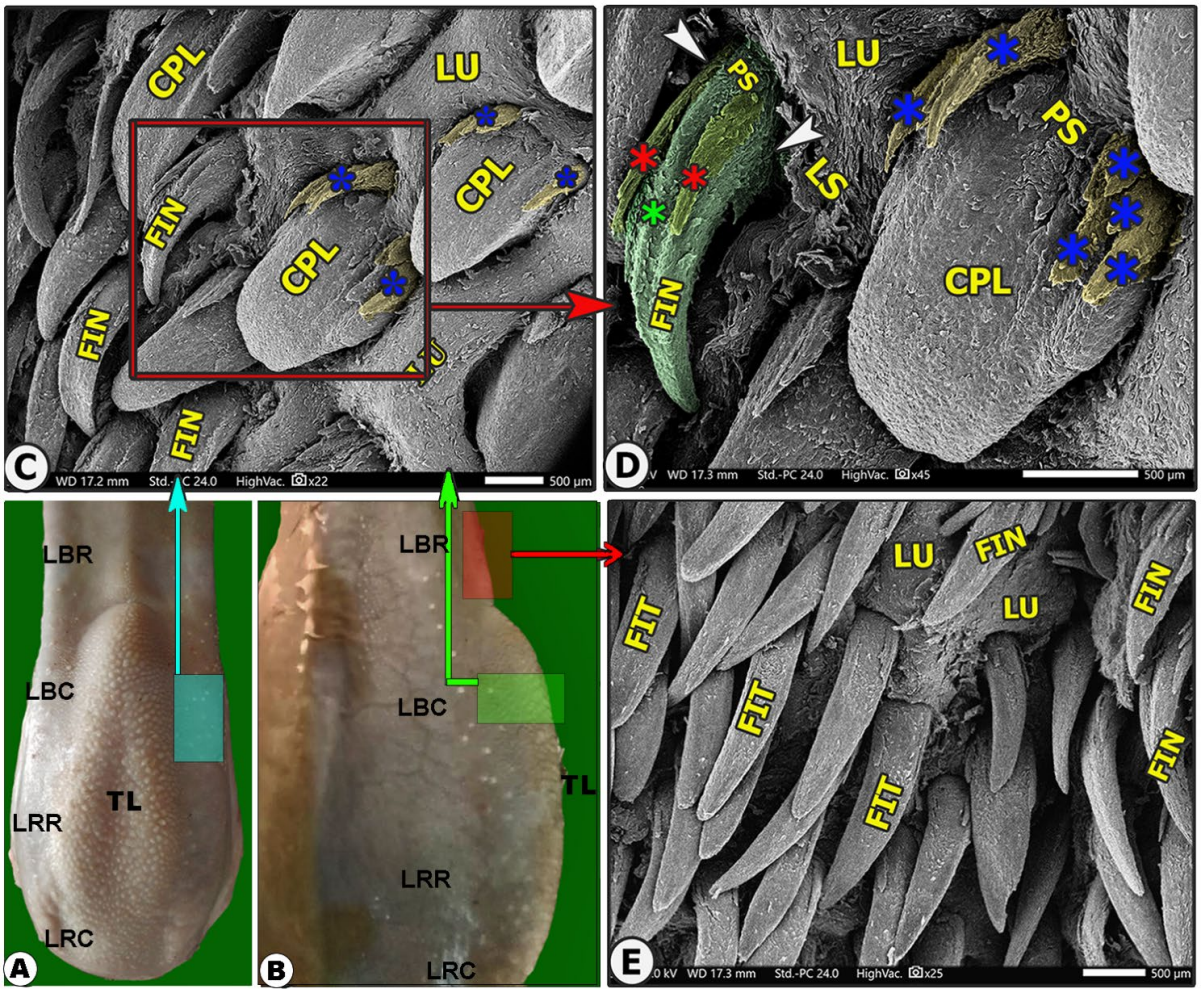
Gross (Views A-B) and scanning electron microscopic (Views C-E) image of the lateral area of the lingual body (LB) of the Arab Zebu cattle to show its rostral (LBR) and caudal (LBC) part; the rostral (LRR) and caudal (LRC) part of the root; torus lingua (TL); tongue-like fiflirom papillae (FIT); transient conical papillae (FIN) with their accessory processes (red*); median papillary ridge (green*); papillary scales (PS); the lingual tubercle (LU); and scales (LS); the large processed conical papillae (CPL) with its accessory processes (blue*). Scale bars: C, D, and E = 500 μm

**Fig. 10:**
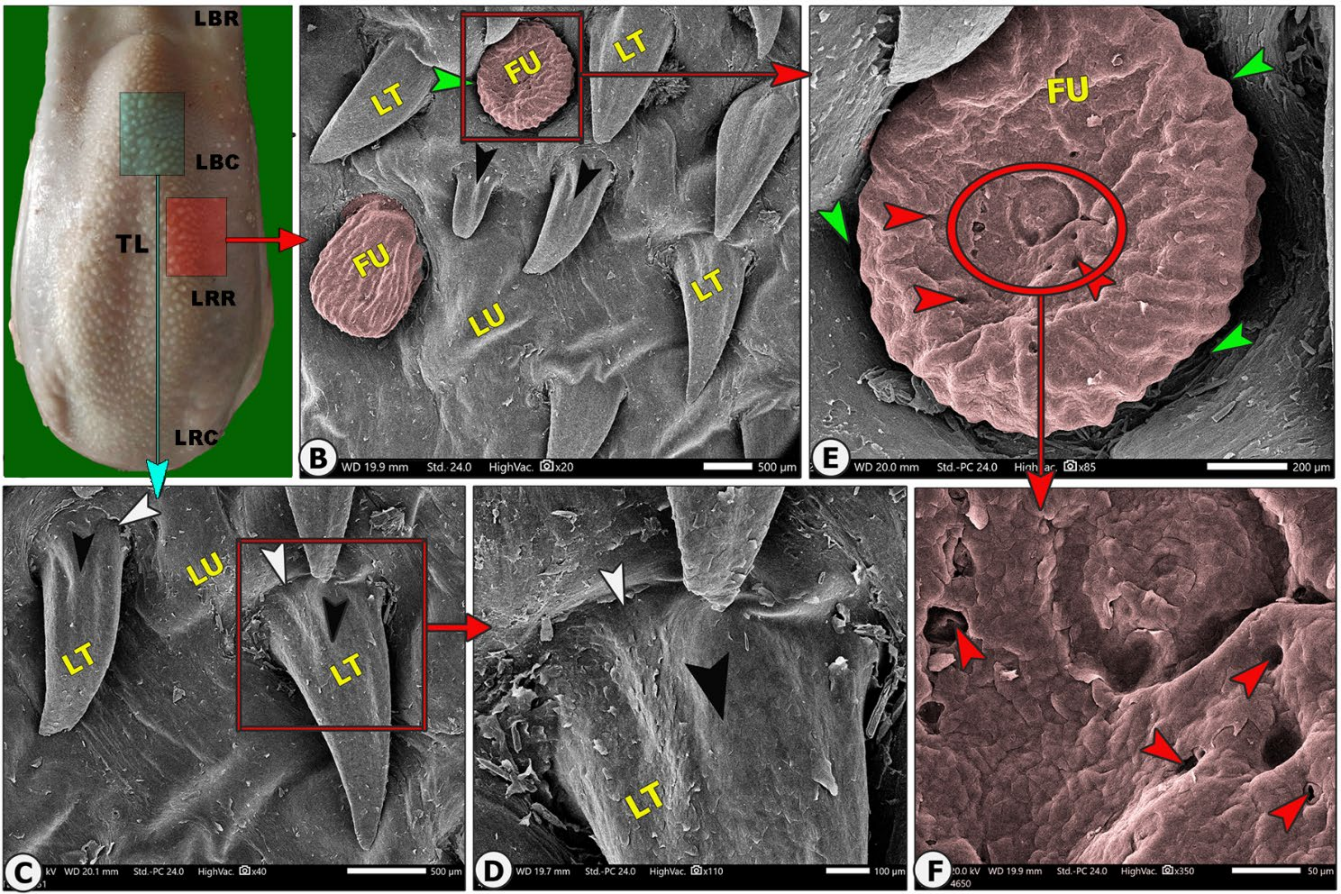
Gross (View A) and scanning electron microscopic (Views B-F) image of the dorsal surface of the torus linguae of the Arab Zebu cattle to show the rostral (LBR) and caudal (LBC) parts of the body, the rostral (LRR) and caudal (LRC) parts of the root, and the torus lingua (TL), lentiform papillae (LT) with their median ridge (black arrowheads) and circular groove (white arrowheads), the fungiform papillae (FU) with their surrounding groove (green arrowheads), and taste pores (red arrowheads). Scale bars: B and C = 500 μm, D = 100 μm, E = 200 μm, F = 50 μm

The lingual papillary system, located on the dorsal and lateral surfaces, consists of mechanical and gustatory types, with mechanical papillae consisting of filiform, conical, and lentiform, and gustatory papillae having fungiform and circumvallate types. The lingual tip’s dorsal surface is covered by numerous keratinized mucosal folds, tubercles, and few filiform papillae (Figs. 2B–E and 3B–E), while the lingual apex and body are covered by densely distributed filiform papillae with few fungiform papillae (Figs. 4B–C, 5B–C, 6B-C and 7B–D, and 8A–C). The torus lingua’s dorsal surface exhibited few lentiform papillae, while its lateral surfaces had few conical papillae with circumvallate papillae, as shown in (Figs. 9C–E and 10B).

### Filiform papillae (FP)

The most abundant caudally directed thread-like filiform papillae were found on the dorsal lingual surface of the apex (excluding its tip) and the rostral part of the body (Figs. 4B-C, 5B-C, 6B-C, 7B-D and 8A-C, and 9C-E), while they were scanty on the tip only (Figs. 2B-E and 3B-E). There were six distinct subtypes of filiform papillae: the long, rod-like, elongated, tongue-like, leaf-like, and transient conical papillae (Figs. 2, 3, 4, 5B–C, 6B–C, 7, 8 and 9C–E).

### Long filiform papillae

These papillae were rare on the lateral area of the dorsal tip surface, while the median and anterior tip surfaces had numerous mucosal folds, tubercles, projections, and scale-like projections (Figs. 2B-D and 3B). These papillae had a circular base surrounded by scales, a pointed apex, and a broad, long body with one pair of pointed accessory processes on each side. Papillary scales were present between these two accessory processes on the dorsal surface (Figs. 2B–D and 3).

### Rod-like filiform papillae

These papillae were found in the caudal area of the tip, just posterior to the median mucosal fold area, and appeared to originate from pores surrounded by numerous scales, as depicted in (Figs. 2B and 3B-E). The papillae appeared to originate from pores surrounded by numerous scales, as depicted in (Fig. 3C-E). The papillae had an ovoid base and a short body that bifurcated into two or three rod-like processes with a pointed apex. The papillae displayed more abundant scales and tubercles than the long filiform papillae (Fig. 3C-E).

### Elongated filiform papillae

They were the most abundant caudally directed thread-like filiform papillae found on the median area of the dorsal surface of the apex and the rostral part of the body, excluding its tip (Figs. 4B-C, 5B and 8A-C). The papillae had a base surrounded by scales, a pointed or round apex, and an elongated body with a median ridge and two pairs of pointed accessory processes on each side (the ventral long one and the dorsal short one). They also had numerous papillary scales between each pair of accessory processes (Figs. 4B-F, 5B-C and 8A-C).

### Tongue-like filiform papillae

They were extensively distributed on the lateral area of the dorsal surface of the apex (excluding its tip) and the rostral part of the body, which were separated from each other by numerous lingual tubercles (Figs. 6B-C and 7B-D), in addition to the scales in the apex only (Fig. 6B). The papillae had a base, round apex, and elongated body with pointed accessory processes. They had numerous papillary scales between the two accessory processes on the dorsal papillary surface (Figs. 6B-C and 7B-D).

### Leaf-like filiform papillae

Their few numbers were observed on each side of the median line of the rostral part of the body and were surrounded by numerous tubercles (Fig. 8C). The papillae had a base, a circular groove, scales, a pointed apex, and an elongated, slightly dorsally curved body with elevated lateral edges forming the groove on the dorsal surface of the papillary body. It had two pairs of papillary pointed accessory processes, one ventral long and one dorsal short, and numerous papillary scales between them (Fig. 8C and E-F). The papillae were found in various morphologies.

### Transient conical papillae

Their few numbers were observed on the dorsal surface of the lateral area of the rostral part of the body, just corresponding to the fossa linguae, and before the area of the large conical papillae on the lateral surface of the torus linguae. Furthermore, these papillae were considered a transient conical form between the tongue-like filiform papillae and the conical papillae (Fig. 9C-E). The papillae had a circular base with a circular groove and numerous scales, a pointed apex, and an elongated, slightly dorsally curved body with a median longitudinal papillary ridge and one pair of papillary pointed accessory processes (one on each side). Furthermore, between the two accessory processes, there were a few papillary scales (Fig. 9C-E).

### II. A. 2. Conical papillae (CP)

**Fig. 11:**
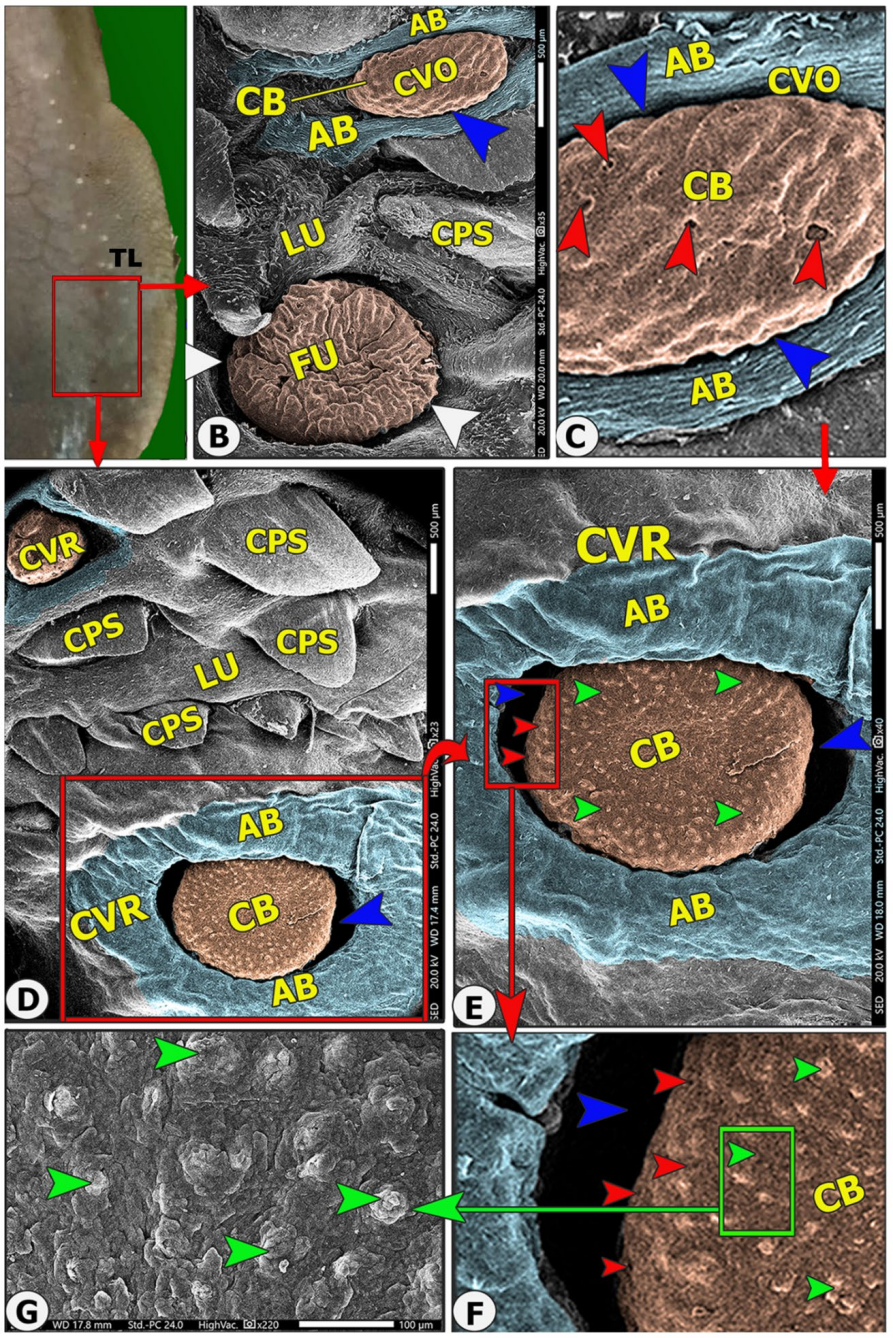
Gross (View A) and scanning electron microscopic (Views B-F) image of the lateral surface of the torus linguae of the Arab Zebu cattle to show; the torus lingua (TL), small conical papillae (CPS), lingual tubercle (LU), fungiform papillae (FU) with therir surrounded groove (white arrowheads), round (CVR) and ovoid (CVO) circumvallate papillae with papillary body (CB), annular bad (AB), annular groove (blue arrowheads), taste pores (red arrowheads), and papillary tubercles (green arrowheads). Scale bars: B, D, and E = 500 μm, C and F = 200 μm, G = 100 μm

**Fig. 12:**
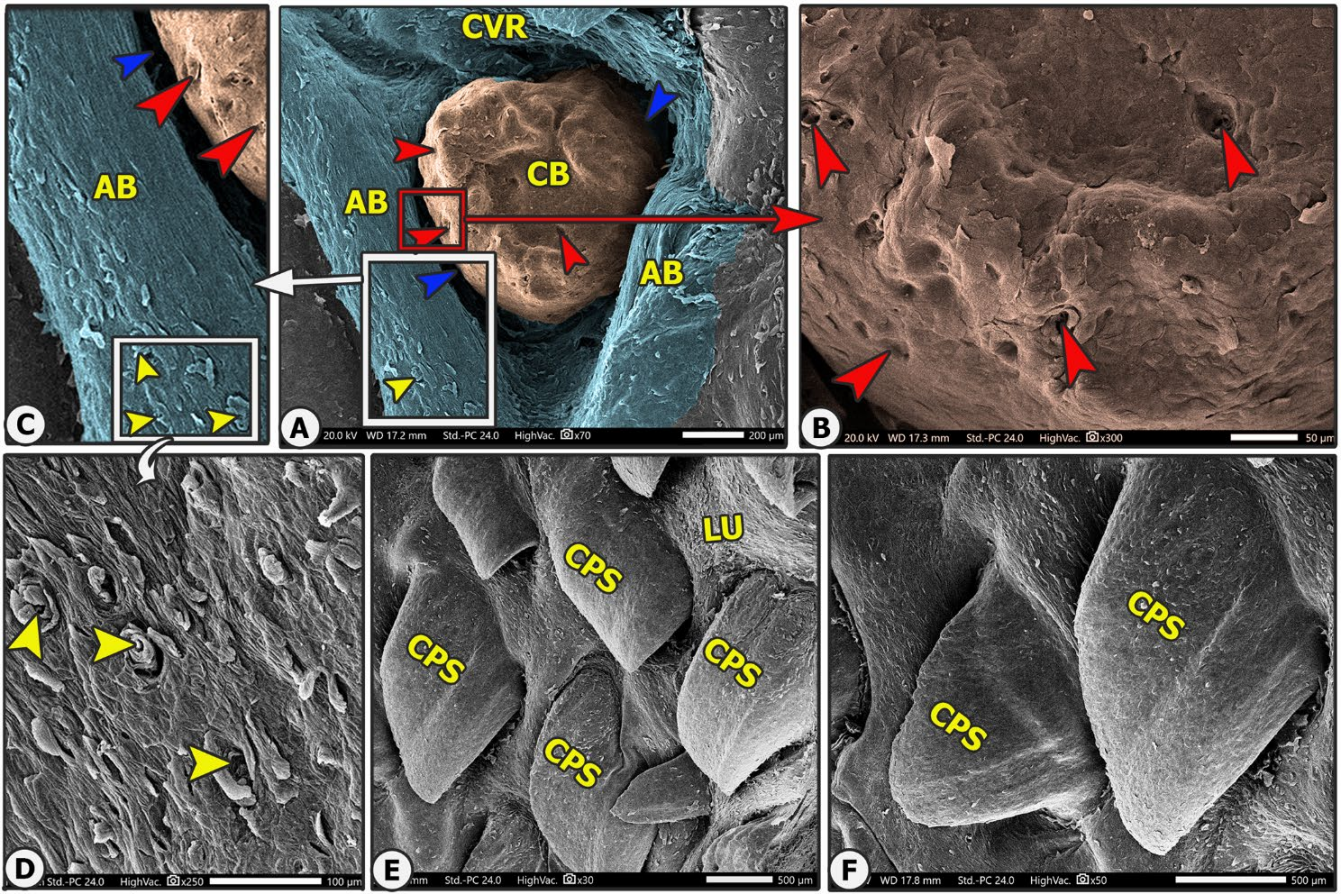
Scanning electron microscopic image of the lateral surface of the torus linguae of the Arab Zebu cattle to show the small conical papillae (CPS), lingual tubercle (LU), round circumvallate papillae (CVR) with papillary body (CB), annular bad (AB), annular groove (blue arrowheads), taste pores (red arrowheads), and papillary scales (yellow arrowheads). Scale bars: A = 200 μm, B and C = 50 μm, D = 100 μm, E and F = 500 μm

Conical papillae were found on the lateral surface of the torus linguae and had a wide, semicircular base and a rounded, blunt apex. They can be subtyped into large processed and small conical papillae (Figs. 9C-E and 11B and D, and 12E-F).

### Mechanical papillae

The mechanical lingual papillary system comprised filiform (FP), conical (CP), and lentiform (LFP) papillae that had a caudal direction towards the pharyngeal cavity, as described in (Figs. 2A-B, 3B-E, 4B-F, 5B-C, 6B-C, 7B-G, 8, 9C-E, 10B-D and 11D, and 12E-F).

### Large processed conical papillae

They were observed on the lateral surface of the rostral part of the torus linguae, which was located just in front of the transient conical papillae. Each papilla had a wide base with four pairs of accessory papillary processes (four on each side) with a slightly curved body and a blunt round end, as well as a few papillary scales observed on the dorsal papillary surface between the accessory papillary processes (Fig. 9C-E).

### Small conical papillae

They were observed on the caudal portion of the lateral surface of the torus linguae that surrounded the region of the circumvallate papillae and were separated from each other by the lingual tubercles. These papillae consisted of a wide base, a slightly curved body, and a blunt end (Fig. 11D, and 12E-F). They outnumber the large papillae in number.

### Lentiform papillae (LFP)

Elongated triangular papillae were found on the dorsal surface of the torus linguae, among a few of the fungiform papillae and lingual tubercles. These papillae consisted of a circular base that was surrounded by a circular groove and a slightly curved body with a median longitudinal ridge and a pointed apex (Fig. 10B-D).

### Gustatory papillae

There were two types of the gustatory papillae; the fungiform (FU) and circumvallate (CV) papillae.

#### Fungiform papillae (FU)

Generally, scanty fungiform papillae were observed on the dorsal and lateral surfaces of the tongue. The fungiform papillae had two papillary subtypes: oval and round papillae, with each papilla bordered by a circular groove and possessing tasting pores. The *oval fungiform papillae* were observed on the dorsal surface of the tip (among the mucosal folds and a very few long filiform papillae) and the lateral portion of the dorsal surface of the apex and rostral part of the body (among the tongue-like filiform papillae), while the *round fungiform papillae* were observed on the dorsal surface of the torus linguae among the lentiform papillae; moreover, each papilla was surrounded by a papillary groove (Fig. 10B and E, and 11B).

The study found that each *oval fungiform papilla* was surrounded by a groove (Figs. 2B-C and F and 3B, and 6C-F), with 3–5 taste pores and papillary scales on its dorsal surface, as shown in SEM magnification (Fig. 2F, and 6C-F). Each *round fungiform papilla* was surrounded by a groove (Fig. 10B, and 10E), and its corrugated dorsal surface had numerous folds and 5–9 taste pores, as observed in SEM magnification (Fig. 10E–F). The fungiform papillae on the rostral part of the body were found to carry taste-bad and hair-like processes (Fig. 6C-F).

#### Circumvallate papillae(CV)

On the two lateral surfaces of the caudal wide part of the torus linguae (rostral papillary region of the root); there were 25–27 pairs of circumvallate papillae in two longitudinal rows (dorsal and lateral), with 13–14 papillae in each dorsal row and 12–13 papillae in each ventral row. There were two subtypes of circumvallate papillae: round and ovoid papillae. Each papilla consisted of a papillary bulb that was encircled by an annular groove and a vallum (Fig. 11B and D-E, and 12A). The vallum of the ovoid circumvallate papillae was U-shaped (Fig. 11B), while the round circumvallate papillae were completely encircled by the annular bad (Figs. 11D-E and 12A). The *round papillary bulb* carried 8–14 taste pores on their dorsal and lateral papillary surfaces (Fig. 11E-F, and 12A), while the *ovoid papillary bulb* carried 6–10 taste pores on their lateral surface corresponding to the annular groove (Fig. 11C). The annular border was covered in scales and projected lingual scales at high SEM magnifications (Fig. 12A and C).

### Morphometric SEM analysis

**Fig. 13:**
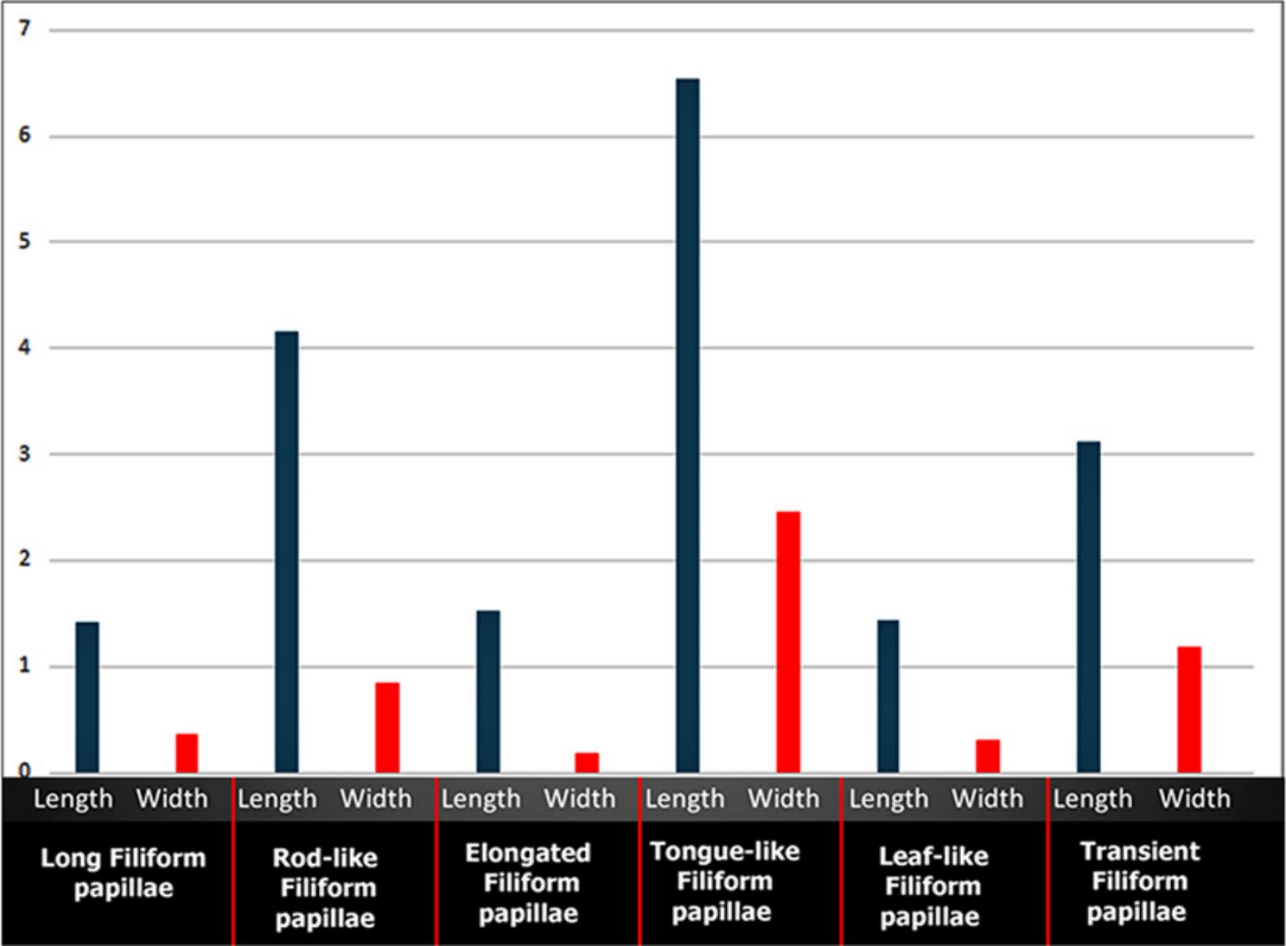
Graphic chart showing the different average dimensions (length and width) of the six different subtypes of the filiform papillae on the dorsal lingual surface of the apex and body

**Fig. 14:**
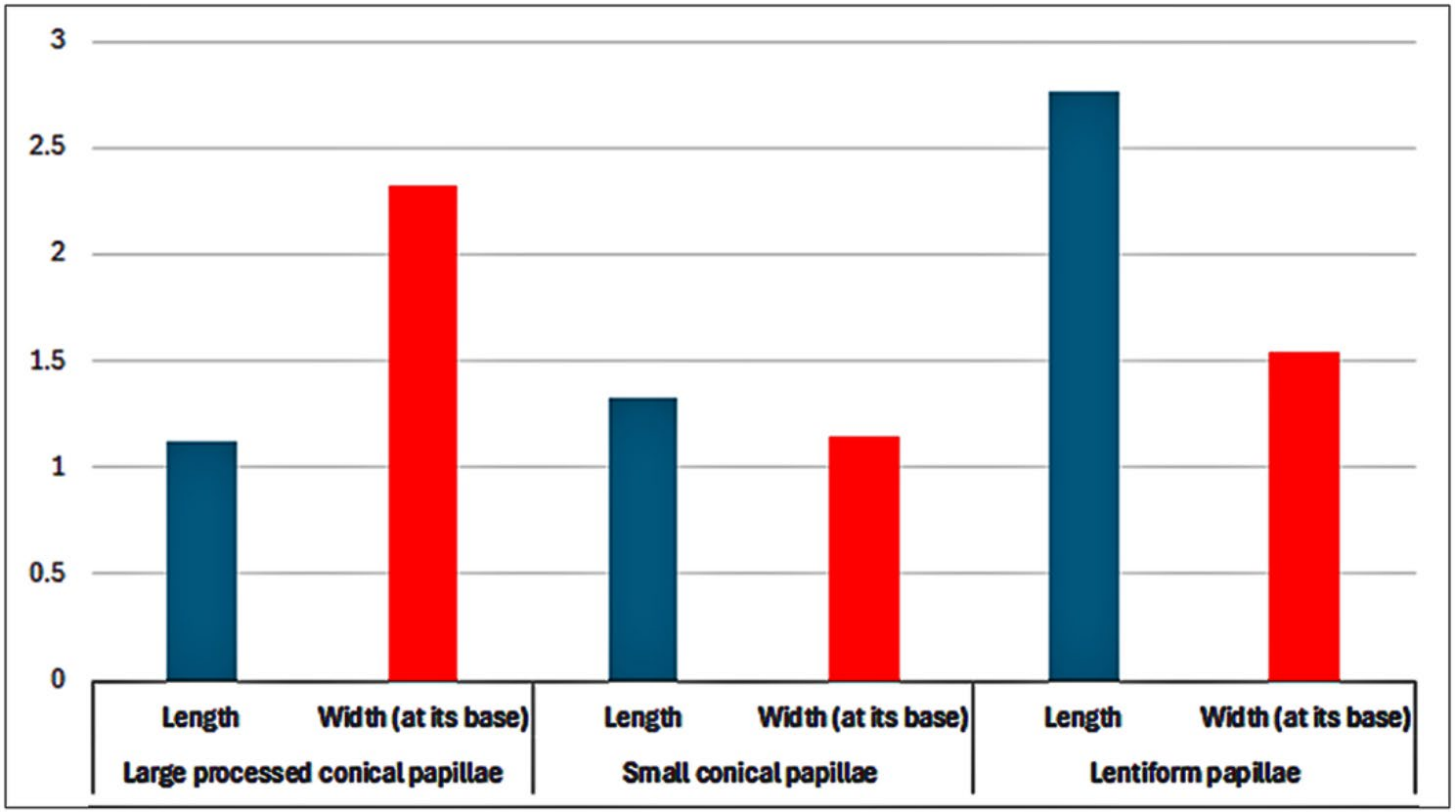
Graphic chart showing the different average dimensions (length and width) of the two subtypes of the conical papillae (on the lateral surface of the torus linguae) and lentiform papillae (on the dorsal surface of the torus linguae)

The average length and width of the different six subtypes of the filiform papillae with their accessory processes on the dorsal and ventral lingual surfaces were described in (Fig. 13). The tongue-like filiform papillae were the longest (6.543 ± 2.21 μm) with the widest base (2.453 ± 0.563 μm), followed by the rod-like filiform papillae that had an average length of (4.154 ± 0.543 μm) and an average width of (0.842 ± 0.032 μm). The shortest filiform papillae were the long filiform papillae (1.418 ± 0.23 μm) and leaf-like filiform papillae (1.432 ± 0.564 μm). Meanwhile, the elongated filiform papillae (0.187 ± 0.021 μm) were the less wide papillae (Fig. 13). The average length and width of the two subtypes of conical and lentiform papillae were described in (Fig. 14). Furthermore, the average diameter of round fungiform and circumvallate papillae, as well as the major and minor axes of oval fungiform and ovoid circumvallate papillae, was described in (Table 2).

**Table 2:** Shows the average dimensions of the different papillary subtypes of the fungiform and circumvallate papillae on the dorsal surface of the arab Zebu cattle Tongue

Gustatory papillae		(um)
**Oval fungiform papillae** **(On the dorsal surface of the lingual tip and the lateral portion of the apex and rostral part of the body)**	**Major axis** **Minor axis**	0.876 ± 0.32 0.513 ± 0.41
**Round fungiform papillae** **(On the dorsal surface of the torus linguae)**	**Diameter**	1.765 ± 0.75
**Round Circumvallate papillae** **(On the lateral surface of torus linguae)**	**Diameter**	2.533 ± 0.57
**Ovoid Circumvallate papillae** **(On the lateral surface of torus linguae)**	**Major axis**	1.765 ± 0.08
	**Minor axis**	0.754 ± 0.23

## Discussion

The present study is designed to describe the SEM features of the lingual papillary system of Arab Zebu cattle in Chad, which live in the Savanna Forest, a harsh ecological desert area in the Sahel [2]. The cattle rely on dry, hard-textured herbs, which may contain thorns or rough extrusions, to provide nutrition. This study is the first to describe their environmental adaptations to the Savanna Forest. Our findings reveal numerous lingual adaptations in Arab Zebu cattle, including numerous mucosal folds and tubercles, along with a few filiform and fungiform papillae on the lingual tip, which have not been previously described in any animal species. Our study reveals that the unique characteristics of the lingual tip suggest that the cattle studied did not rely on food particle selection patterns due to the scanty availability of food particles. The current study reveals the presence of well-developed filiform papillae on the dorsal surface of the apex (excluding the tip) and rostral body part, which aid in fixing captured food particles and preventing them from escaping the oral cavity. Additionally, this study found that the lingual tip has relatively scanty papillae, lacking the usual papillae present in other areas of the tongue and those present on the lingual tip of other ruminant species [6, 9, 14, 16–19]. This suggests that cattle may have a specialized mechanism for manipulating food particles with precision. The observed papillae on the tip, as well as the mucosal folds and tubercles, and the small number of fungiform papillae, were not previously described in other ruminant species.

Our gross investigation illustrated that Arab Zebu cattle have a three-part elongated tongue (apex, body, and root), torus linguae, and fossa linguae, comparable to what has been found in other ruminant species [6, 9, 14, 16–19]. Furthermore, our findings revealed that the presence of a slightly elevated median ridge on the dorsal surface of the body was not previously recorded in any ruminant species, whereas most ruminant species had a median lingual groove on the dorsal lingual surface of the apex [6, 7]. Functionally, the median lingual ridge aids in the fixation of hard-textured food particles and prevents them from escaping the oral cavity. Previous SEM reports suggest that lingual papillae morphological changes are primarily linked to dietary habits, strategies, natural and environmental conditions [8, 20]. Different lingual papillary types in animal species have varying topography based on food particle availability, shape, size, orientations, micro-architecture, and nomenclature and are divided into mechanical and gustatory types based on their role in food selection and processing [6, 9, 20]. Lingual papillae, as described in our study, and other ruminant species such as buffalo, goat, and sheep [6, 14, 20], are classified into five types based on their mechanical and gustatory functions: mechanical (filiform, conical, and lentiform) and gustatory (fungiform and circumvallate). Ruminant species like barking deer and Bactrian camels have four types of lingual papillae: two mechanical (filiform and conical) and two gustatory (fungiform and circumvallate) [21, 22]. The five varieties of lingual papillae with specific-region distribution, shape, size, and directions, as well as the numerous lingual tubercles, scales, and mucosal folds of the examined Arab Zebu cattle, play a significant role in feeding patterns adapted to the Chad environmental conditions.

The filiform papillary system, a species- and region-specific trait in all animal species, is a key indicator of an animal’s lingual adaptation to its feeding mechanism and environmental conditions, influenced by its shape, size, number, orientation, organization, and nomenclature [9, 23]. Our SEM analysis revealed that the tip of the apex has few filiform papillae, while well-developed filiform papillae are present on the dorsal surface of apex and rostral part of the body. These lingual papillary modifications were matched with the presence of very dry, hard-textured herbs in Chaian Savanna Forest, which may contain some thorns or abrasive extrusions of the Arab Zebu cattle. In contrast, the filiform papillae only cover the rostral motile lingual region in all animal species, including ruminants [20, 21, 24].

Filiform papillary subtypes exist in various animal species, are influenced by feeding habits and mechanisms, and are classified based on available food particles and environmental factors like geographical dispersion and population ecological conditions [6, 9, 14, 20]. Filiform subtypes are believed to significantly influence the capture mechanism of nutritional material particles, their fixation in the buccal cavity, and their orientation towards the pharyngeal cavity [14, 20]. Our study revealed six subtypes of the complicated filiform papillary system in Arab Zebu cattle: long, rod-like, elongated, tongue-like, leaf-like, and transient conical papillae. Notwithstanding, large domesticated ruminants like camels and Egyptian water buffalo have only one filiform papillae subtype [14, 25], while small ruminants with harsh environmental conditions feeding like the Egyptian Ossimi sheep tongue have five subtypes: ventral and dorsal processed, triangular, leaf-like, and triangular processed filiform papillae [20], but other small ruminants like alpaca and llama, sheep, and goat have two filiform subtypes [6, 17, 26, 27]. Some non-ruminant grass-eating animals, like rabbits [28], have three filiform subtypes, while carnivorous red foxes have five [29]. These filiform papillary subtypes are the most common lingual structures altered to accommodate different feeding methods [6, 14, 30].

Our description of accessory papillary processes on the surface and scales in between was related to dry spinated food particles found in Chad, in which these secondary accessory papillary processes arise from the base of the five subtypes of the filiform papillae. Our findings found that these accessory papillary processes are found in various positions of the lingual regions; on the lateral area of the dorsal surface of the tip and apex of the filiform papillae; on the rostral part of the body of the tongue-like filiform papillae; on the dorsal surface of the lateral area of the body of transient conical papillae; on the dorsal surface of the median line of the rostral part of the body of the leaf-like filiform papillae; on the dorsal lingual surface of the apex and the rostral part of the body of the elongated filiform papillae (had two pairs; the ventral long one and the dorsal short one). The observation of the papillary basal origin of these secondary accessory processes is similar to that of some ruminant species in that respect [21, 24]. Secondary accessory processes are present in all filiform subtypes, except for the triangular filiform papillae in the Egyptian Ossimi sheep tongue, which does not carry these processes [20]. Ruminant species have varying numbers of secondary processes of the filiform papillae, with sheep having 1–3 pairs [17], Egyptian goats having 3 pairs [6], goats having 3–4 pairs [31], Saanen goats having 2–3 pairs [24], and lesser-mouse deer having 1–2 [32]. In the Egyptian Ossimi sheep tongue, there are three pairs of accessory processes on the dorsal surface of each filiform papilla, while two papillary processes are present on each ventral processed filiform papilla [20].

Papillary subtypes are present in mechanical conical papillae, but to a lesser extent than in filiform papillae. Our study identifies two conical papillary subtypes on the torus linguae’s lateral surface: small and large processed conical papillae, with the large processed conical papillae carrying four pairs of accessory secondary papillary processes. Two conical subtypes are found in other small ruminants but with different nomenclatures, such as the Egyptian Ossimi sheep tongue, which has large ones (on the torus linguae’s dorsal surface) and small ones (on the torus linguae’s lateral surface) [20], while the Egyptian goat tongue has two subtypes: large curved and small straight papillae [6]. Some ruminants, like humped camels, goats, lesser-mouse deer, and Formosan serow, have only one type of conical papillae [24, 32–34], while Turkish sheep and Bactrian camels have completely absent conical papillae. The conical papillae, on the other hand, were completely absent in Turkish sheep and Bactrian camels [17, 22]. Our study found triangular-pointed lentiform papillae on the dorsal surface of the torus linguae, similar to those found in other ruminants [6, 14, 20, 27, 30]. Conversely, the lenticular papillae in the torus linguae of deer, Formosan serow, and sand mazama species were found to be entirely absent [21, 34–36]. The study by [17] identified the existence of two papillary subtypes of mechanical lentiform papillae on the torus linguae in sheep: the small bifid papillae on the rostral part of the dorsal surface of the torus linguae and the long ones on the caudal part of the torus linguae. Functionally, the conical and lentiform mechanical papillae in ruminants fix nutritional food material during mastication to compensate for deficient dental structure.

The gustatory fungiform papillae are categorized into three types based on their function, distribution, and morphology. Fungiform papillae are typically gustatory, containing taste pores or buds, as found in most mammals [6, 7, 20, 28]. However, they can also be mechanical papillae without taste pores or buds, as seen in Saanen goats and donkeys [24, 37], and mixed fungiform papillae (some papillae have taste pores and others do not), as seen in horses and cows, providing a fascinating description [38]. Our research identified two subtypes of Egyptian endemic small ruminants: round and oval, based on papillary shape, similar to those found in goats and sheep [6, 20]. Most mammals have one type with different shapes, such as mushroom papillae in Saanen goats [24], dome papillae in the raccoon dogs and foxes [39], and round papillae in the pampas deer [40]. The classic distribution of fungiform papillae in most animals is among the filiform papillae to better protect them [14, 24]. The fungiform papillary distribution had some species-specific features to aid in gustatory function. Our study described that the fungiform papillae were very scanty on the dorsal and lateral lingual surfaces, in which the oval fungiform papillae were observed on the dorsal surface of the tip (among the mucosal folds and a few long filiform papillae) and the lateral area of the dorsal surface of the apex and rostral part of the body (among the tongue-like filiform papillae), while the round fungiform papillae on the dorsal surface of the torus linguae were among the lentiform papillae. Numerous fungiform papillae were found on the dorsal and ventral surfaces of the lingual tip; similar findings were found in Egyptian Ossimi sheep [20], goats [6], European bison, cattle, Bison bonasus hybrids [41], fallow deer [42], and Egyptian water buffalo [14].

The previous SEM data on the fungiform papillary surface showed minor variations, particularly in the number of taste pores. Our SEM magnifications revealed that round fungiform papillae have corrugated dorsal surfaces with numerous papillary folds and 5–9 taste pores, while oval papillae have 3–5 taste pores and papillary scales on their peripheral border; additionally, the fungiform papillae on the rostral part also carry taste pores and hair-like processes. The Egyptian Ossimi sheep tongue’s fungiform papillae carried numerous taste pores on their dorsal surface, particularly those found on the torus linguae [20], in which in the Egyptian Ossimi sheep, the ovoid papillae carried 5–10 taste pores by an especial projected-like papillae, while the round papillae on the apex and body have 10–15 taste pores by an especial projected-like papillae, and the ones observed on the torus linguae carried 20–25 taste pores by the projected-like papillae [20], in which this appearance of the taste pores on especial projected-like papillae has not been previously described in any ruminant species. The tongues of various animals, including the Formosan serow, one-humped camel, Bactrian camel, yak, and sheep, have 2–3 taste pores on their fungiform papillae [17, 22, 30, 33, 34]. The current study aligns with previous research on ruminants, revealing the circular groove around fungiform papillae [22, 33, 34, 36]. Papillary grooves are absent in pampas deer and Egyptian Ossimi sheep [40], while the hair-like processes described in our study are also found on the dorsal fungiform papillary surface [20].

According to previously published articles, the circumvallate papillae vary from numerous in ruminants [6, 7, 20, 26] to totally absent in the cape hyrax and Guinea pig [43, 44]. Circumvallate papillae are found on the two lateral surfaces of the torus linguae in most ruminants, including the currently examined cattle [6, 14, 20, 21, 32, 36, 45]. Our study reveals two subtypes of circumvallate papillae on the lateral surface of the torus linguae: round and ovoid, which are varied in some points; firstly, the annular band is U-shaped in ovoid papillae and circular in round ones, while the round papillae have 6–10 taste pores on their dorsal and lateral surfaces, and ovoid papillae have 6–10 taste pores on their lateral surface corresponding to the annular groove. The Egyptian Ossimi sheep tongue has one type of ovoid circumvallate papillae with 2–5 taste pores [20]. The presence of taste buds facing the papillary groove was observed in Egyptian goats [6], one-humped camels [33], Egyptian water buffalo [14], and deer [21].

The annular groove and bad around each circumvallate bulb are common in most ruminants, including Egyptian water buffalo [14], the sheep [20], goats [6, 24], and alpaca [46]. The Egyptian Ossimi sheep tongue has two annular pads, U-shaped external pads with three parts not fused anteriorly, and ovoid internal pads with one layer encircling the annular groove [20]. Most ruminants, like sheep, lambs, goats, Egyptian water buffalo, and cattle, have a single annular pad [6, 14, 17, 24, 38, 47], while Barbary sheep lack an annular groove [48]. In the one-humped camel, there are two or three circumvallate papillae, bordered by primary and secondary papillary grooves [33]. In chital deer and sheep, some circumvallate papillae are not surrounded by the annular pad and groove [17, 19], while in the dromedary camel, one annular pad is surrounded by two or three papillae [33]. Functionally, these annular pads organize the arrival and detention of salivary secretion in the annular groove [17].

Circumvallate papillae number varies among animals, particularly abundant ruminant species, and is influenced by their feeding method. The study found a high number of circumvallate papillae, which compensate for the limited presence of fungiform papillae, with 25–27 pairs on the two lateral surfaces of the caudal wide part of the torus linguae in two longitudinal rows (dorsal and lateral), with each row having 13–14 papillae. The Egyptian Ossimi sheep’s tongue has 12–13 pairs of papillae on the lateral surface of the caudal part of the torus linguae in two longitudinal rows, with the dorsal row having 12–13 pairs and the ventral row having 11–12 pairs [20]. Meanwhile, the Turkish sheep had 6–10 papillae [17], Saanen goat had 13–14 papillae [6, 24], Egyptian water buffalo had 10–12 papillae [14], the cattle had 11–16 papillae [30], cattle-yak had 14 papillae [45], chital deer had 11–14 papillae [19], and barking deer had 10–13 [21]. In contrast, non-ruminant herbivorous animals have fewer circumvallate papillae, primarily located on the dorsal surface of lingual roots, such as the Nile grass rat, which has only one median-located circumvallate papillae [49], but the rabbit has two lateral circumvallate papillae [28], while the Egyptian long-eared hedgehog has three circumvallate papillae [49]. Our high SEM magnifications revealed that the dorsal surface of circumvallate papillae was corrugated, identical to Egyptian Ossimi sheep, Iranian goats, cattle-yaks, and alpacas [20, 26, 27, 45], whereas the smooth papillary surface was found in sheep [17].

## Conclusion

The Arab Zebu cattle’s lingual papillary system, as observed in SEM, appears to be adapted to Chadian environmental conditions. The dorsal surface of the apex and rostral parts has well-developed filiform papillae, while the tip has few. The torus lingua’s dorsal surface has few lentiform papillae and two conical papillae subtypes. Six filiform papillae subtypes were identified, including long rod-like, tongue-like, and transient conical/leaf-like ones. Two fungiform papillae subtypes were found to be surrounded by a groove and have taste pores. The U-shaped annular bad and circular bad were observed around ovoid circumvallate papillae, with regional divergence specialized for their harsh and semi-harsh diets.

## Data Availability

The datasets used and/or analyzed during the current study are available from the corresponding author on reasonable request.

## Abbreviations

SEM: Scanning Electron Microscope
FP: Filiform papillae
CP: Conical papillae
LFP: Lentiform papillae
FU: Fungiform papillae
CV: Circumvallate papillae
